# Editorial: RAS inhibitor therapy of cancer

**DOI:** 10.3389/fonc.2025.1685295

**Published:** 2025-09-09

**Authors:** Donald J. Buchsbaum, Nicolas N. Nassar, Gary A. Piazza

**Affiliations:** ^1^ Department of Obstetrics and Gynecology, University of Alabama at Birmingham, Birmingham, AL, United States; ^2^ Department of Radiation Oncology, University of Alabama at Birmingham, Birmingham, AL, United States; ^3^ Department of Pediatrics, University of Cincinnati College of Medicine, Cincinnati, OH, United States; ^4^ Division of Experimental Hematology & Cancer Biology, Signaling & Drug Discovery Program, Cincinnati Children’s Hospital Medical Center, Cincinnati, OH, United States; ^5^ Department of Drug Discovery & Development, Cancer Research Center, Auburn University, Auburn, AL, United States

**Keywords:** RAS, RAS inhibitors, RAS inhibitor therapy, combination therapy with RAS inhibitors and chemotherapy, combination therapy with RAS inhibitors and immunotherapy, combination therapy with RAS inhibitors and other targeted therapies, MAPK inhibitors

## Background

RAS signaling plays an essential role in normal cellular proliferation, while aberrant activation of this pathway is a driver of multiple cancers. KRAS is the most frequently mutated oncogene in human cancers, present in about 30% of cancers, with high frequencies in lung adenocarcinomas (32%), colorectal carcinomas (50%), and pancreatic cancers (95%). Mutations in other RAS genes also contribute to human cancers, for example, NRAS mutations are common in melanoma, while HRAS mutations frequently occur in head and neck cancers. Amplification and activation of non-mutated wild-type (WT) RAS is a feature of other cancers such as esophageal, stomach, ovarian, testicular cancers and of neurofibromatosis type 1 (NF1).

KRAS-G12C inhibitors have recently received FDA approval, which represent a breakthrough in the development of targeted therapeutic strategies against RAS oncogenic proteins previously considered undruggable. The KRAS-G12C inhibitors sotorasib (AMG510) and adagrasib (MRTX849) are being used to treat patients with KRAS-G12C-mutated non-small cell lung cancer, although colorectal cancer patients with the same mutation appear to be resistant. Recent research shows that other point mutations in KRAS can be targeted by small-molecule inhibitors. KRAS inhibitors have shown promising results in clinical trials. For example, Mirati Therapeutics developed a KRAS-G12D inhibitor, MRTX1133, which showed significant preclinical antitumor activity in KRAS-G12D-tumor bearing mice, especially pancreatic ductal adenocarcinoma. However, major limitations for allele-specific KRAS inhibitors are the heterogeneity of KRAS mutations, coexistence of multiple KRAS mutations, and resistance that is caused by the emergence of new KRAS mutations or from activation of co-expressed wild-type RAS isozymes by upstream signaling pathways. As such, pan-RAS inhibitors, and inhibitors of SOS1 are being developed that show promise in preclinical studies and the potential for greater efficacy or reduced potential for resistance. Alternatively, combining a RAS inhibitor with other drugs might overcome resistance.

We originally announced the Research Topic “*RAS Inhibitor Therapy of Cancer*” in *Frontiers in Oncology* in 2023 to invite manuscripts devoted to investigations of the mechanisms, selectivity, efficacy, toxicity, and drug resistance of RAS-targeted inhibitors for the treatment and hopefully cure of cancer. We were interested in articles describing therapeutic advances using RAS-targeted inhibitors administered alone or in combination with other molecularly targeted drugs, chemotherapy, radiation therapy, or immunotherapy. This editorial summarizes the 5 peer-reviewed and published articles on this Research Topic.

## Summary of published articles

Since the recent FDA-approval of covalent KRAS G12C inhibitors for the treatment of non-small cell lung cancer, there have been intense efforts by the pharmaceutical industry and university investigators to develop new RAS inhibitors by targeting additional mutant forms of the KRAS protein for broader applications to treat cancers driven by other KRAS mutations, especially KRAS G12D. Additional approaches already in clinical trials involve both pan-KRAS and pan-RAS inhibitors to address efficacy limitations arising from secondary mutations and acquired resistance. The review by Lokhandwala and colleagues provides an overview of ongoing research involving both covalent and non-covalent approaches to directly inhibit RAS from the lens of a structural biologist (Lokhandwala et al.).

Initial monotherapy trials of both sotorasib and adagrasib demonstrated a stark contrast in efficacy between CRC and NSCLC. In the Phase I CodeBreak 100 trial, sotorasib achieved an objective response rate (ORR) of 36% in KRAS G12C-mutant NSCLC, with responses lasting over 6 months in most responders. By comparison, in KRAS G12C-mutant metastatic CRC (mCRC), sotorasib monotherapy yielded only a 9.7% ORR. Adagrasib likewise, showed robust activity in NSCLC but similar low single-agent efficacy in CRC, underscoring a tissue specific disparity. Preclinical and translational data point to multiple, overlapping resistance mechanisms that limit monotherapy activity in CRC. Secondary KRAS alterations, upstream and downstream MAPK node mutations, RTK-SHP2 mediated feedback, epithelial-mesenchymal transition (EMT), alternate proliferative axes, and proteostasis deregulation are among the intrinsic and acquired mechanisms of resistance highlighting why CRC cells adapt more readily than NSCLC to G12C-targeted blockade. To forestall or reverse resistance, multiple combination regimens are under active investigation (Piazza et al.).

KRAS G12C inhibitor monotherapy had limited clinical efficacy for KRAS G12C- mutated colorectal cancer due to primary and acquired resistance mechanisms. Combinations of KRAS G12C inhibitors with other targeted therapies, such as RTK, SHP2, and MEK inhibitors, have been investigated in clinical trials to overcome the resistance. Efficacy was demonstrated especially by combining KRAS G12C and EGFR inhibitors. Combinations of KRAS G12C inhibitors with other targeted therapies, such as SOS1, ERK, CDK4/6, and wild-type RAS, are ongoing in clinical trials. Preclinical data identified additional promising KRAS G12C combinations with YAP/TAZ-TEAD inhibitors, FAK inhibitors, and farnesyltransferase inhibitors. The combinations of KRAS G12C inhibitors with immunotherapies and chemotherapies have also been reported. Combining KRAS inhibitors with other therapeutics is expected to play a role in future treatment of KRAS-mutated cancers (Miyashita et al.).

There is an urgent unmet medical need to develop novel KRAS inhibitors for pancreatic cancer as KRAS is mutated in over 90% of patients diagnosed with pancreatic cancer. Unfortunately, FDA-approved KRAS G12C inhibitors have limited impact on this most fatal malignancy given the diversity of KRAS mutations that give rise to the disease as well as their role in driving resistance to mutant-specific KRAS inhibitors. Nonetheless, such drugs have paved the way for a new generation of KRAS inhibitors in clinical trials that address the complex mutational landscape of pancreatic cancer. The review by Long et al. provides an overview of preclinical research and clinical trials aimed at developing new KRAS inhibitors for the treatment of pancreatic cancer (Long et al.).

RAS/MAPK pathway mutations are found in a subset of non-GIST soft tissue sarcomas (STS) and are enriched in hypermutated tumors. Standard management relies on cytotoxic chemotherapy regimens with or without radiotherapy. Single targeted agents, mainly inhibitors of angiogenesis and of cell cycle have shown activity in only specific subtypes. MEK inhibition has shown limited clinical benefit outside of NF1-mutant tumors. To overcome pathway redundancy and adaptive feedback, combinations of MAPK-inhibitors with driver-mutation blockade, with RAF-inhibitors, and with VEGFR- and PI3K/AKT-inhibitors may broaden response in hypermutated STS. Given the heterogeneity of non-GIST STS, comprehensive genomic profiling and a better characterization of the immune and stromal microenvironments will allow better personalized treatments (Al Jarroudi et al.).

